# Recruiting patients cost-effectively by mail

**DOI:** 10.1186/1745-6215-12-S1-A117

**Published:** 2011-12-13

**Authors:** Theingi Aung, Helen Cowan, Richard Haynes, Louise Bowman, Jane Armitage

**Affiliations:** 1 Clinical Trial Service Unit, University of Oxford, Oxford, OX3 7LF, UK

## Introduction

Large randomised trials have been successfully conducted using mailed drug supply and follow-up [1-4] . ASCEND (A Study of Cardiovascular Events iN Diabetes) is a randomised “2x2 factorial design” study of aspirin versus placebo and of omega-3 fatty acid versus placebo, for the primary prevention of cardiovascular events in people with diabetes. In order to be able to study 15,000 people with diabetes for about 7 years at low cost, ASCEND is streamlined and run mainly by mail with back-up from a 24-hour Freefone service.

## Methods

In collaboration with consultants around the UK, potentially eligible people with diabetes have been identified from various sources including: centrally-held registers (e.g.: Retinopathy screening registers); GP-held local registers and self-referral via a website. For patients identified from centrally-held registers, permission was gained from the National Information Governance Board to allow centrally generated letters in the name of the holder of the register, to be sent to patients listed on registers. In addition, with the collaboration of the Diabetes Research Network and the Primary Care Research Network, 730 general practices agreed to send pre-assembled invitation packs to people on their locally held registers.

To facilitate recruitment, the design is straightforward with simple inclusion and exclusion criteria and treatment packaged for easy mailing. Double sided A3 forms are used for screening, randomisation and follow-up. On the Screening form, patients confirm their eligibility and consent that they are happy to take part by ticking a series of boxes. If they have any questions then they can telephone study staff via a Freefone number. Screening forms of those entering the study are checked centrally by clinical staff. Patients enter a 2-month pre-randomisation run-in phase and are randomised if they complete a randomisation form and remain willing and eligible.

## Results

In total, 423,286 people with diabetes were invited to take part and 15,481 randomised (see table).

**Table 1:** 

	Centrally-held register	GP practices (Local register)	Others*	Total
Invitations sent	300,188	120,875	2223	423,286
Patients enter run-in	16104	9,741	635	26480
Patients randomised	9013 (3.0%)	6038 (5.0%)	430 (19.3%)	15481 (3.7%)

* HPS follow up/Self/friends/Hospital referral

## Conclusion

If sufficient numbers of potentially eligible patients can be identified centrally and trial treatments require little in the way of monitoring, the recruitment and follow-up of patients in clinical trials by mail is feasible and cost-effective. Wider use of these methods could allow more large randomised trials to be undertaken successfully and cost-effectively.
